# A Compact and Low Cost Electronic Nose for Aroma Detection

**DOI:** 10.3390/s130505528

**Published:** 2013-04-25

**Authors:** Miguel Macías Macías, J. Enrique Agudo, Antonio García Manso, Carlos Javier García Orellana, Horacio Manuel González Velasco, Ramón Gallardo Caballero

**Affiliations:** 1 University Center of Merida, University of Extremadura, Sta. Teresa de Jornet, 38, Mérida 06800, Spain; E-Mail: jeagudo@unex.es; 2 Polytechnic School, University of Extremadura, Cáceres 10003, Spain; E-Mails: antonio@nernet.unex.es (A.G.M.); horacio@nernet.unex.es (H.M.G.V.); ramon@nernet.unex.es (R.G.C.); 3 Faculty of Science, University of Extremadura, Avda. Elvas s/n, Badajoz 06006, Spain; E-Mail: carlos@nernet.unex.es

**Keywords:** electronic nose, microcontroller, neural network

## Abstract

This article explains the development of a prototype of a portable and a very low-cost electronic nose based on an mbed microcontroller. Mbeds are a series of ARM microcontroller development boards designed for fast, flexible and rapid prototyping. The electronic nose is comprised of an mbed, an LCD display, two small pumps, two electro-valves and a sensor chamber with four TGS Figaro gas sensors. The performance of the electronic nose has been tested by measuring the ethanol content of wine synthetic matrices and special attention has been paid to the reproducibility and repeatability of the measurements taken on different days. Results show that the electronic nose with a neural network classifier is able to discriminate wine samples with 10, 12 and 14% V/V alcohol content with a classification error of less than 1%.

## 1. Introduction

During the last four decades a lot of research has been directed towards developing olfactory electronic systems or electronic noses. During this time, automatic odour detection systems have been applied in many industrial applications in agriculture [1], indoor air quality [2], environmental monitoring [3], quality control of food products [4,5], medical diagnosis [6], as well as many others.

Some developments have been directed towards the construction of low cost and compact electronic noses [7], which have led to a number of commercial e-nose products [8]. However, they are still too expensive for widespread use and are usually closed systems that do not allow the user to change the hardware. This means that, for example, the sensors in the commercial electronic noses may not be changed for specific applications.

Current microcontrollers allow products and devices to be controlled easily and quickly. The mbed NXP LPC1768 Microcontroller [9] is a single-based board 32-bit microcontroller that is designed for prototyping all sorts of devices as well as a teaching tool somewhat along the lines of Arduino [10,11]. This platform is also the first commercially available solution to fully implement the cloud-based model for embedded systems programming on real hardware [12] and permits many types of applications, such as use in the control of residential electricity [13], in educational CANSAT's rocket for aerospace education [14], to develop a behavioural-based robot platform [15] or in a self-balanced personal transporter robot platform [16].

Microcontrollers cover a high range of applications like automotive control systems, implantable medical devices, remote controls and toys. The use of microcontrollers for E-Nose systems is not new [7,17–19], microcontrollers allow portability, connection with the different sensors and prototyping possibilities. However in some cases [7,20,21] you need an external PC or PDA with a data acquisition card to collect or process the data and complete the system. In addition, if you want to use a microcontroller you need to understand the microcontroller architecture and the specific instruction set and assembler code in order to start to work. In the case of the mbed microcontroller you have a powerful ARM-based tool that allows you to create autonomous applications with lots of interfaces to connect with and an easy to learn architecture programmable in C++, which means a project can be developed with limited knowledge of electronics and programming.

In this paper, we report on the development of a portable e-nose system prototype using an mbed microcontroller that allows, in a very simple way, control of the operation of the electronic nose, data storage and communication with the user during the measurement process. This design allows the research community to have a very low cost electronic nose (around $US 200) that can be configured and used with a very basic knowledge of electronics. Finally, the performance of our electronic nose has been tested by choosing between synthetic wine samples (aqueous ethanol solutions) with different ethanol content 10, 12 and 14% V/V.

Section 2 describes the electronic nose hardware and the general characteristics of the mbed microcontroller. In Section 3 we test the viability of our olfactory system in the detection of the ethanol content of synthetic wine matrices and, finally, in Section 4 the results and conclusions of the project are presented.

## 2. The Hardware

### 2.1. The MBED Microcontroller

**Figure 1.**
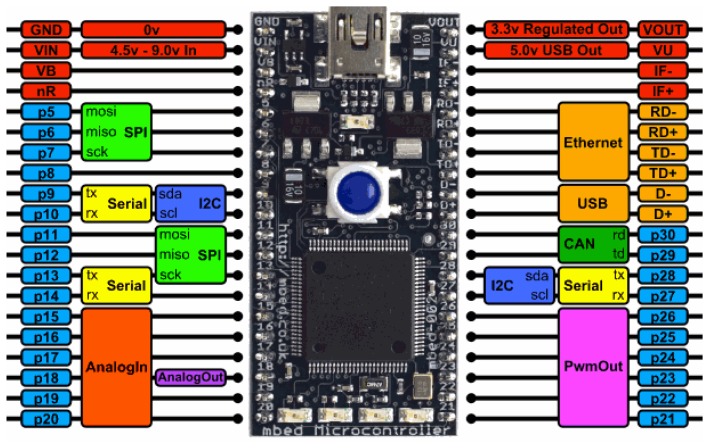
Mbed pinout [9].

The setup and development of a microcontroller platform is often difficult, especially more powerful controllers. Even expensive tools require a lot of time and training to operate correctly. NXP helps those who need to work with ARM controllers: the mbed module comes with online development tools. The module, 44 mm × 26 mm, contains an LPC1768 ARM7 microcontroller and a USB port. It can also include an Ethernet connection, the flexibility of lots of peripheral interfaces and FLASH memory. It is packaged as a dual in-line package for prototyping with through-hole PCBs and includes a built-in USB FLASH programmer. This last characteristic is one of the most important because it means neither an external programmer nor removal of the mbed is necessary to program or update an application, it can be done through the USB port. The NXP LPC1768 includes an ARM Cortex-M3 core running at 96 MHz. It includes 512 KB FLASH, 32 KB RAM and lots of interfaces: built-in Ethernet, USB Host, USB Device, CAN, SPI, I2C, ADC, DAC, PWM among others. In Figure 1, the pinout and the commonly used interfaces and their locations can be seen.

The FAT filesystem feature is one of the keys to easy use. Creating a new program for the device is simply a matter of copying the binary file to this drive. Once copied, only the reset button needs to be pressed and the new program is copied to the internal flash and run. Neither a special programming hardware dongle nor special bootloader software are needed, just drag and drop.

The programming philosophy of mbed is based on the cloud computing concept. The mbed microcontroller's programs are hosted by the *mbed.org* developer website, including a lightweight online compiler for instant access to your working environment through a web browser and therefore possible on any OS. One can move between home and office and resume editing code on any system. There is no need to check for software updates, as the server will always be current. Mbed programs are written in C++ and the environment includes a C/C++ SDK for productive high-level programming of peripherals. Combined with a complete set of libraries and code examples published by the mbed community, the platform provides a productive environment for achieving results.

### 2.2. The Proposed Electronic Nose System

Measurements with our e-nose are taken using the dynamic headspace technique [22]. In dynamic headspace sampling, the headspace of the vials is continuously swept into the detector by a clean purge flow for analysis. This way, the gaseous analyte concentration immediately above the liquid phase is kept as low as possible to increase the evaporation rate. This evaporation rate depends on the surface area, the analyte surface concentration, the analyte volatility and the sample temperature.

**Figure 2.**
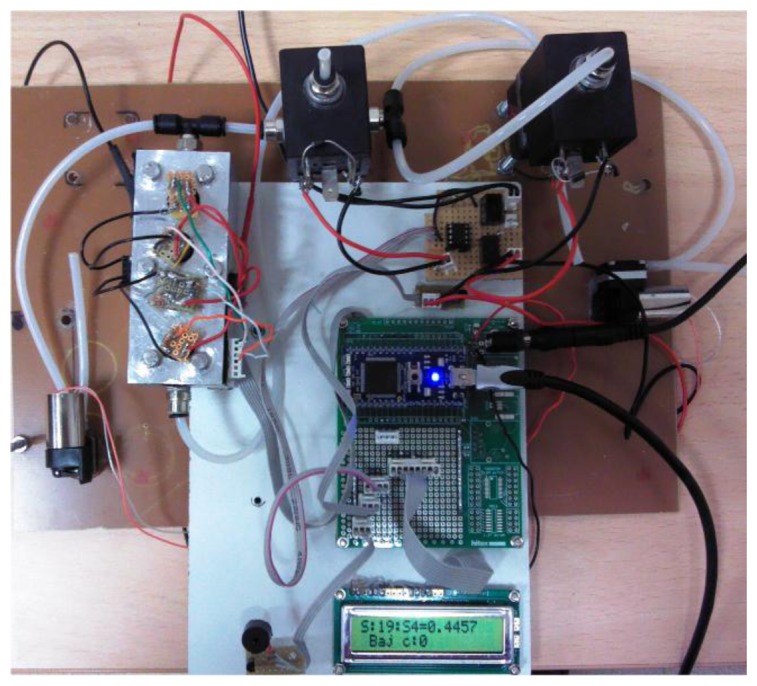
View of the mbed electronic nose.

To do this, the electronic nose hardware is composed of two small pumps, two electro-valves and a sensor chamber which are connected and controlled by the mbed. A view of the mbed electronic nose can be observed in Figure 2.

**Figure 3.**
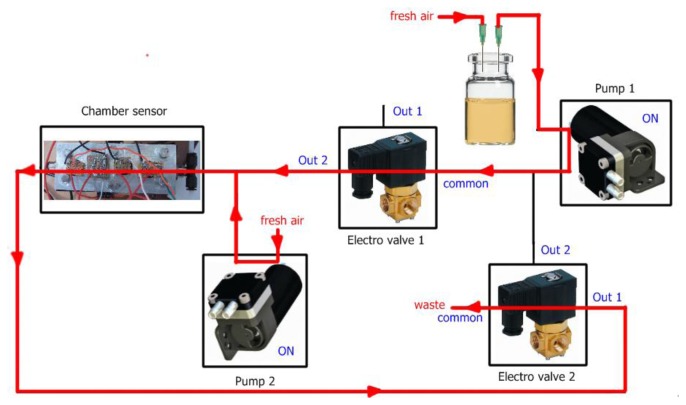
Flow of gases at the injection stage.

**Figure 4.**
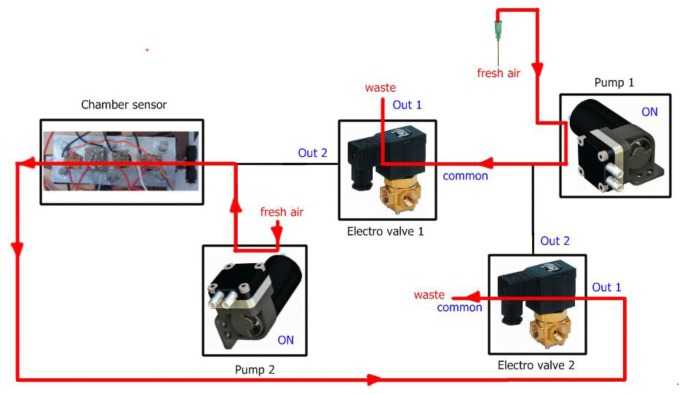
Gases flow at the cleaning1 stage.

**Figure 5.**
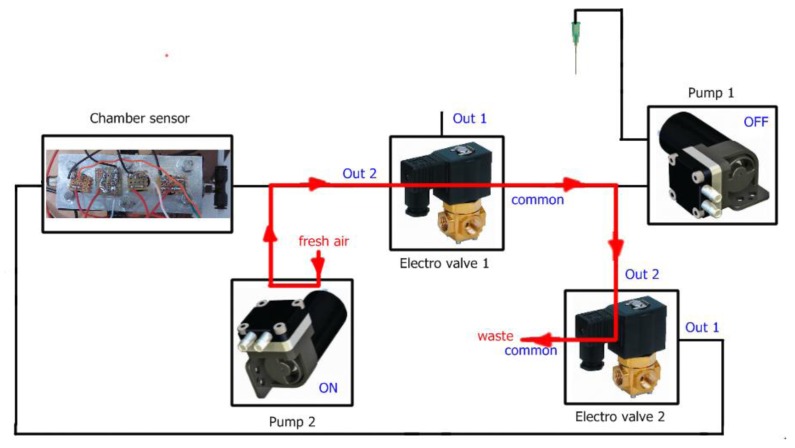
Flow of gases at the cleaning2 stage.

Each measurement with our electronic nose is divided into two stages, *injection* and *cleaning*. In the injection phase the volatiles of the sample (carried by pump 1) mixed with fresh air (carried by pump 2) are transported to the sensor chamber to be analyzed by the array of sensors. In the cleaning phase all traces of volatiles must be removed from the electronic nose to avoid interference with the next measurement. The voltage applied to the pumps controls the amount of gas flow and the electro valves control the path of the gases. In addition, to ensure the cleaning of the entire system, the cleaning stage is divided in two substages *cleaning1* and *cleaning2*. The flow of gases in the injection and cleaning stages can be observed in Figures 3, 4 and 5 respectively.

This project uses four TGS Figaro gas sensors, TGS26XX (with XX = 02,11,00,20) obtained from Figaro Engineering, Inc. (Osaka, Japan). These sensors show a certain degree of affinity towards a specific gas but are sensitive towards a wide spectrum of gas types with overlapping sensitivities. These sensors have been mounted in a stainless steel sensor chamber. A load resistor is connected in series with each sensor and a common power supply of 5 V is used for both the heater voltage of the sensors and the voltage applied to the voltage divider formed by the sensors and the load resistances. Finally, the response of the sensor is the voltage measured at the output of the voltage divider.

Additionally, a SHt71 temperature and humidity sensor has been installed in the sensor chamber. This sensor has a relative humidity accuracy of ±3.0%RH and a temperature accuracy of 0.4 °C. It operates from a voltage supply in the range of 2.4 V and 5.5 V and is connected to an I^2^C bus on the mbed microcontroller.

Fresh air is used as carrier gas and measures start when the sensor resistances in the presence of the carrier gas are stabilized. Before a new measurement the value of the initial voltage output (V_o_) is calculated as the mean value of the measurements made during an interval of time called the *set time*. Then, in the cleaning and injection stages, measures are taken at intervals of time, the *measurement interval*, and the value of the output voltage of the sensor divided by the initial voltage (V/V_o_) is saved in a *measurement file*.

### 2.3. Working Modes

The electronic nose can operate in two different ways, the measurement mode and the analysis mode. In the measurement mode a *measurement configuration file* is read from the USB o SD card and a *measurement file* with the measurements of the sensors is written in the USB. These measurement files can be used in a PC to train a neural network that can be used in a subsequent analysis phase. In the analysis mode a neural network previously trained is used to make the analysis of the new measurements. In this case two configuration files must be read a *measurement configuration file* and another file containing information about the parameters of the PC's previously trained neural network in the *neural network configuration file*.

Regardless of the operation mode, a new measurement always starts reading the *measurement configuration file* that contains the following parameters defined in the previous section:

- Number of sensors
- Cleaning1 stage time
- Cleaning2 stage time
- Injection stage time
- Set time
- Pump 1 voltage
- Pump 2 voltage
- Measurement interval

### 2.4. The MBED Configuration

The pins of the mbed are configured as follows:

- p15–p20: Analog inputs to read the voltage of the sensors.
- p5–p14: Control the LCD display.
- p27–p28: Control the sh71 temperature and humidity sensor that communicates with the microcontroller by the bus I^2^C.
- p21–p22: PWM out to control the gas flow of pumps 1 and 2. The amount of gas flow is controlled by the duty cycle of the PWM signal.
- P23: PWM out to control the sound alarm that warns the user of the beginning of a new injection stage.
- P24–p25: Digital outs that control the two electro valves
- P31–p32: Control the USB mass storage device to save the measurement files and the configuration files of the electronic nose.

The mbed also includes a network environment and a remote procedure calls over web sockets that allow the user to control the electronic nose via the internet, which can be very useful in high risk applications.

## 3. The Electronic Nose Test

The alcohol content of some synthetic wine samples was quantified in order to test the electronic nose. To do this three synthetic wine samples were prepared, aqueous ethanol solutions at 10, 12 and 14% V/V and twenty measures of each one were taken, so 60 prototypes were classified in three different classes and labeled as *alc10*, *alc12* and *alc14*. Each prototype is contained within a file with the measurements of the four sensors during the injection and cleaning phases.

Moreover, given that the big problems faced while working with electronic noses are reproducibility and repeatability, measures were taken on different days and, especially, prototypes measurements of the different classes were taken alternatively. If the measurements are not taken in this way, the ability to separate classes could be due to sensor drift or instability, among other causes, rather than the alcohol content of the sample.

After numerous tests, measurements were taken with the following values of the adjustable parameters of the electronic nose:

- Number of sensors: 4
- Cleaning1 stage time: 400 seconds
- Cleaning2 stage time: 50 seconds
- Set time: 10 seconds
- Injection stage time: 2 seconds.
- Pump 1 voltage: 100% duty cycle (200 mL/min).
- Pump 2 voltage: 10% duty cycle (20 mL/min).
- Measurement interval: 0.25 seconds.

**Figure 6.**
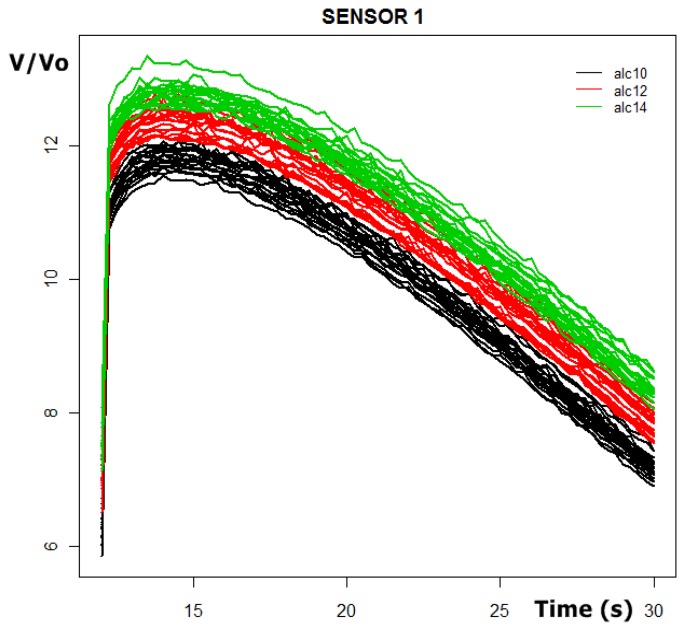
Sensor 1 response curves for the 60 prototypes.

In Figure 6 we can observe the sensor response curves of sensor 1 for the 60 prototypes in the 12–30 seconds interval of time.

### 3.1. Feature Extraction

During the measurement time of the electronic nose and depending on the sampling period, each response curve is composed of N points. A lot of features have been proposed for the characterization of sensor response curves: maximum, minimum, slope, average, etc. In this project, the use of principal component analysis (PCA) [23] is proposed to reduce the dimensionality of the N-dimensional vector that initially characterizes the sensor response.

**Figure 7.**
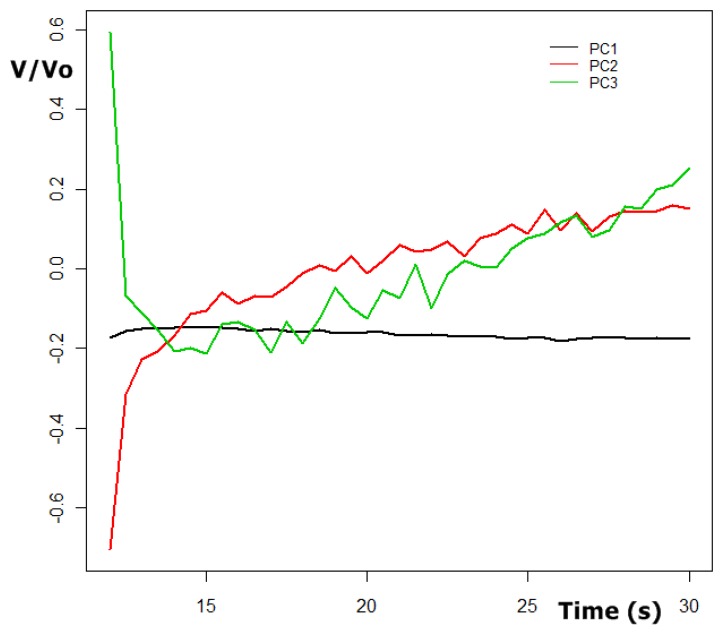
First three principal components of the PCA.

The original purpose of PCA was to reduce a large number of variables to a much smaller number of new ones, the principal components (PCs), whilst retaining the variation in the original variables as much as possible. If we consider the sensor's response curves in figure one there are 60 curves of 56 points each (28 seconds and a sampling interval of 0.5 seconds) therefore there is a data matrix of 60 rows and 56 columns. Applying PCA to the data matrix the principal components were obtained. The first three autovectors of the covariance matrix can be observed in Figure 7, and according to PCA, each initial response curve of Figure 6 can be approximated by a linear combination of these auto vectors or basis functions. The coefficients of this linear combination (the first principal components) form the new variables representative of each response curve.

In Table 1, the proportion of the variance of the first three principal components can be observed. In this case, only the first PC accumulates the 98.1% of the initial variance, and therefore this component was only used to characterize each response curve. This allows us, hereinafter, to represent each sensor response curve with a one-dimensional characteristic vector, the first principal component.

Through using PCA as we have described above and by inspecting the four sensors response curves we have extracted six features that form the initial characteristic vector representative of each measurement of the electronic nose. These parameters can be observed in Table 2.

In addition, as will be described in Section 3, the best classification results are obtained with a subset of this initial characteristic vector.

### 3.2. The Classification Systems

Hidden layer perceptrons (MLP) and support vector machines (SVM) were used to resolve the classification problem. Neural networks were trained with the package *amore* and SVM with the package *e1071*, both from the free software environment for statistical computing and graphics R (The R project for statistical computing) [24].

The k-fold cross-validation method [25] was used in order to assess the performance of our classification systems in practice. One round of k-fold cross-validation involves partitioning our sample of data into k complementary subsets, from which k-1 subsamples are used to fit our predictive model, and the other subsample (the test set) is used to test its performance. In addition, to achieve a good generalization capability, the set used for adjusting the neural models is divided into a training set and a validation set. The training set is used to train the systems and the validation set is used to choose the best model when a minimum of the error over this set is reached.

In the neural network case, for each experiment one hidden layer perceptron was used where the number of neurons of the hidden layer varied from five to 30 neurons in steps of 5. To avoid local minimum each training process was repeated 10 times randomizing the initial weights. Finally, from all these experiments, the neural network model with the minimum error over the validation set was returned and its performance was measured calculating the error over the test set.

In the SVM case a radial basis is used as a kernel function and the validation set is used to adjust the smoothing parameters of the model, *C* and *γ*. These parameters took values of *2^x^* with *x* varying from −5 to 10. Finally, to reduce variability, multiple rounds of cross-validation were performed using different partitions, and the test results were averaged over the rounds.

## 3. Results

**Figure 8.**
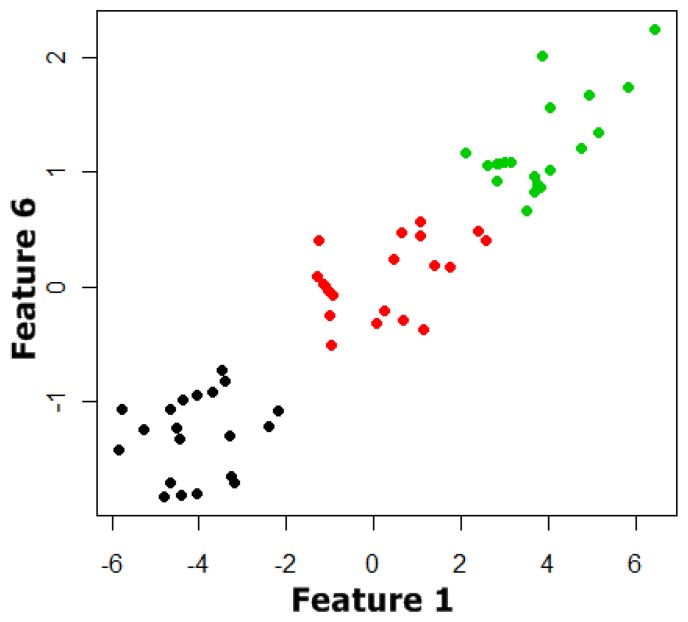
Two dimensional representation of the classification problem.

**Figure 9.**
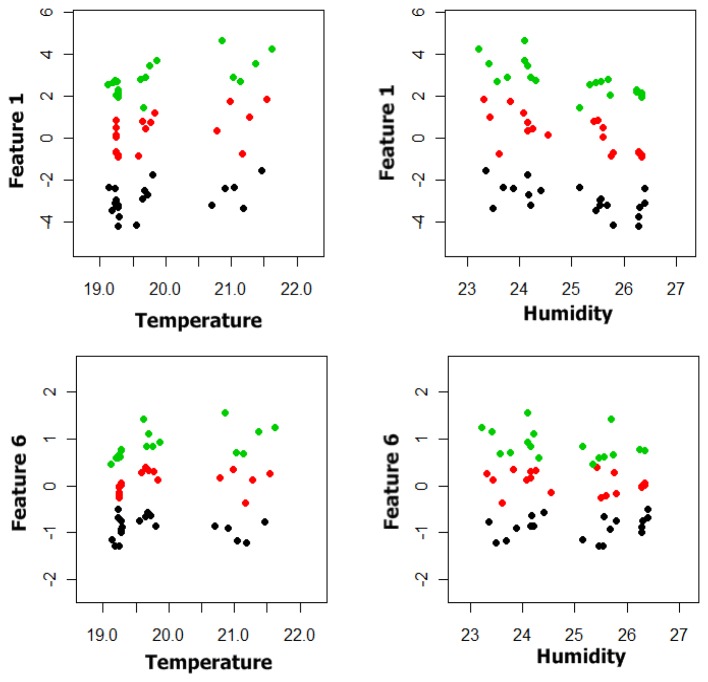
Features 1 and 6 versus temperature and humidity.

Finally, we had 60 prototypes and three classes with each prototype characterized by an initial six dimensional vector. Appling the classification systems as described previously, the best classification results were obtained by using a subset of the initial characteristic vector formed by Features 1 and 6. In Figures 8 and 9 the plot of Feature 6 *versus* Feature 1 and the plot of these features versus the temperature and humidity respectively can be observed. In Figure 9 the data shows that there is not a clear dependency of Features 1 and 6 on the temperature and humidity, so no correction of the initial data has been made.

**Figure 10.**
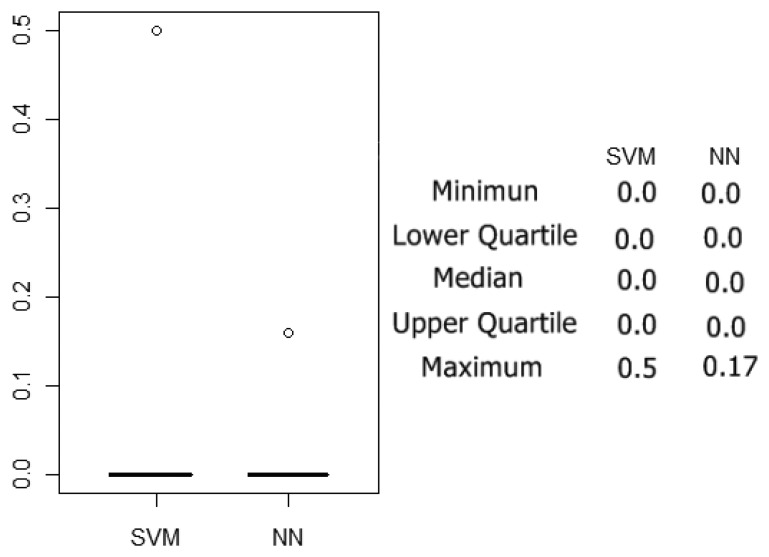
Boxplot of the error calculated over the test sets.

The classification results were estimated using a 10-fold cross validation method and performing 10 rounds of cross-validation, and 100 neural network models and 100 SVMs were tested. The box plot of the errors over the test sets of the 100 simulations previously mentioned can be summarized in Figure 10. In Table 3, the confusion matrix of the 100 neural networks and SVMs generated applying 10 rounds of 10-fold cross-validation is shown. Each one of the 100 networks and 100 SVM was tested over one test set of six prototypes, so 600 individual classifications were made in each case. It can be observed that correct classifications were over 93.5% in the SVM case and 99% in the SVM case. On the other hand, the whole process of training and testing the 100 models lasted over three hours in the SVM case and four days in the NN case.

## 4. Conclusions

We have built a versatile and low cost system that allows an e-nose prototype to be created for a cost of only around $US 200. An mbed microcontroller, two micro pumps, two electro-valves and a chamber sensor (apart from connectors) are all that is needed to start research in the e-nose system field. In addition, the sensors can be easily interchanged or new ones added and it is easy to program and configure the complete system thanks to the mbed microcontroller. The mbed microcontroller has been shown to be a powerful tool for prototyping thanks to its different interface capabilities, easy to use and the need for only limited knowledge of electronics and programming as well as high extensibility.

The results show that this e-nose is comparable with other commercial ones and that it is a valid tool for research requiring a low cost e-nose. The prototype has been tested with three synthetic wine samples, aqueous ethanol solutions at 10, 12 and 14% V/V. NN and SVM classification algorithms were used with good classification results for both, but better for NN. In the NN case correct classifications are 100% for the alc10 and alc14 classes and 99.5% for the alc12 class.

## Figures and Tables

**Table 1.** Importance of the first three components.

	**Standard deviation**	**Proportion of Variance**
PC1	2.90	0.981
PC2	0.28	0.008
PC3	0.19	0.004

**Table 2.** Definition of the characteristic vector representative of the measurements.

**Feature**	**Description of the feature**
1	Projection of the sensor 1 response curve at the interval 10–40 seconds onto the first PC
2	Projection of the sensor 1 response curve at the interval 40–70 seconds onto the first PC
3	Projection of the sensor 2 response curve at the interval 10–20 seconds onto the first PC
4	Projection of the sensor 3 response curve at the interval 10–40 seconds onto the first PC
5	Projection of the sensor 3 response curve at the interval 40–70 seconds onto the first PC
6	Projection of the sensor 4 response curve at the interval 10–30 seconds onto the first PC

**Table 3.** Confusion matrix for the neural network (NN) and the SVM.

**NN/SVM**	**Alc10**	**Alc12**	**Alc14**
Alc10	100%/96%	0%/2.5%	0%/1.5%
Alc12	0%/2%	99.5%/95%	0.5%/3%
Alc14	0%/4%	0%/2.5%	100%/93.5%
